# AI MSK clinical applications: spine imaging

**DOI:** 10.1007/s00256-021-03862-0

**Published:** 2021-07-15

**Authors:** Florian A. Huber, Roman Guggenberger

**Affiliations:** Institute of Diagnostic and Interventional Radiology, University Hospital Zurich, Raemistrasse 100, 8091 Zurich, Switzerland

**Keywords:** Artificial intelligence, Spine

## Abstract

Recent investigations have focused on the clinical application of artificial intelligence (AI) for tasks specifically addressing the musculoskeletal imaging routine. Several AI applications have been dedicated to optimizing the radiology value chain in spine imaging, independent from modality or specific application. This review aims to summarize the status quo and future perspective regarding utilization of AI for spine imaging. First, the basics of AI concepts are clarified. Second, the different tasks and use cases for AI applications in spine imaging are discussed and illustrated by examples. Finally, the authors of this review present their personal perception of AI in daily imaging and discuss future chances and challenges that come along with AI-based solutions.

## Introduction

In the last decade, increasing utilization of artificial intelligence (AI) technologies has taken place for optimizing tasks in almost all aspects of daily life, whether they be as simple as calendar optimization and automatic wake-up alarms, or more complex, for example, self-driving cars [1]. The development of AI technologies has also significantly impacted radiology imaging workflows, specifically musculoskeletal (MSK) and spine imaging tasks along the radiology value chain, which encompasses all main components of radiology workflows vulnerable to change [2, 3].

AI was first mentioned by US computer scientist John McCarthy and was introduced more than 50 years ago, at a time when computational technology was in early development [4, 5]. However, increasing public interest has been observed in the past decade, which is primarily caused by both increasing computing power [6] and the broad availability of high-performance processors for personal use [7]. Recently, a number of investigations have developed AI applications with performance equal to, or even superior to, the human reference standard, both in radiology and elsewhere. This may also have fostered the popularity of AI research and technology among the public. For example, an algorithm known as “AlphaGo,” developed by Google DeepMind defeated the world champion in the board game “Go” in 2016, which gained large-scale public attention by the media, far beyond scientific literature [8]. AI as an umbrella term has heavily influenced our daily routine inside and outside of our radiologic work and has the potential to impact MSK imaging routine in a disruptive way. The scale of the disruption may be comparable to, or even greater than, other changes of the past such as the widespread availability of computed tomography (CT), magnetic resonance imaging (MRI), and picture archiving and communication systems (PACS) [9]. However, while new AI technologies have significant potential, they require basic education of the operator, and more importantly, they present novel challenges and risks that must be kept in mind while pushing forward towards new frontiers. This review is targeted specifically at the radiologist dealing with spine imaging, and provides a basic explanation of AI as well as examples of its application. Furthermore, this article highlights future perspectives and challenges, and discusses how these developments may affect every piece of the conceptual value chain in radiology, as proposed by Enzmann [2].

## Basics of artificial intelligence and its application

AI is the broadest umbrella term for a series of computer algorithms that appear “intelligent” to humans and covers a diverse spectrum of algorithms [10, 11].

Currently, the most dominant approach to AI is machine learning (ML) and builds on the principle of learning from data [11]. ML algorithms essentially require input data, e.g., images in the context of radiology. On the output side of an algorithm resides a prediction, for example, a diagnosis. The process of creating a prediction requires learning a dedicated training objective, which can be achieved by different means [12]. The algorithms used generate predictions in this manner are called classifiers.

ML algorithms are often categorized into unsupervised, supervised, and reinforcement learning methods, and each category requires a different type of input data [11]. Specifically, data can be labelled or unlabelled — unlabelled data contains purely the raw information (for example, a CT or MRI scan), whereas labelled data has been annotated or classified into a specific category (for example, a diagnosis or a segmentation has been made on the scan by a radiologist). Unsupervised and reinforcement learning algorithms can operate on unlabelled data, which is beneficial since, in the vast majority of cases, labelling data requires significant time and energy. Recent developments in a subgroup of unsupervised learning (so-called self-supervised learning models) [13] have attempted to use partly labelled data to make predictions; however, the research is still in its infancy. Thus, supervised learning is still the most frequently used ML method in AI practice in general, and specifically for medical applications [11]. Supervised learning uses labelled data, and thus, it requires a professional radiologist or a separate pre-trained ML algorithm to create a labelled dataset. Thus, supervised algorithms are subject to a professional radiologist or expert in the field paving the way and defining limits of conditions for possible ML approaches, such as classification and segmentation jobs.

The output of ML algorithms largely depends on the quality and selection of input features (“garbage in – garbage out” [14, 15]). This is one of the major issues in applying ML for image classification tasks, as it is very difficult to generate the correct labels for images from unprocessed input pixels or voxels [11]. The difficulty of “supervising” this transformation is the most important trigger for the increasing popularity of a new class of ML called deep learning (DL), which aim to mimic more intelligent behavior through encompassing multiple stages, or layers, in an architecture known as a neural network (NN).

DL can be used synonymously as term for algorithms using NNs [11]. NNs usually consist of several different layers, where the depth of a network refers to the number of layers in successive use. The success of these NNs depends on learning hierarchical features and combining the logic behind radiological diagnoses, and hence, these algorithms usually require vast amounts of parameters, often ranging in the order of several millions [11]. Essentially, DL utilizes end-to-end learning with an automatic computation of a result from imaging input data, allowing the learning of even highly nonlinear functions and dependencies in the input data [4]. DL is therefore used for difficult perceptual tasks, such as in medical imaging, in which it outperforms “traditional” ML strategies.

Convolutional neural networks (CNNs) are a class of DL algorithms which aim to mimic the human visual cortex, and which use specific types of layers in order to become invariant to small variabilities in input data (such as slight image transformations), allowing for a generally good adaptability to new data. With respect to CNN architecture, it is commonly accepted to use the novel term Deep convolutional Neural Network (DNN) if a certain depth, i.e., number of layers, is present [16]. One of the most popular CNNs for image segmentation, the U-Net [17], was developed focusing on high accuracy and combines segmentation and spatial information at different resolutions [11, 18].

Texture analysis (TA) is by definition not an application of AI, but a method of quantitative image analysis [19]. However, it has gained increasing importance in assessing and interpreting images in the recent past [19]. TA aims to investigate tissue characteristics in a predefined region or volume of interest that are invisible to the human eye, extracting quantitative features such as size, shape, intensity, or texture from relationships between pixels and voxels. These features are believed to play a role in improving diagnosis and disease monitoring in different pathologies. In general, TA comes along with large volumes of data [20], i.e., big data and usually requires AI-based techniques for data processing in order to predict certain outcomes. This process is also referred to as “Radiomics” and can derive from any modality. However, limited reproducibility and transferability between imaging modalities, institutions, and scanning units have been reported, and thus, TA algorithms are largely affected by study design and other factors (Table 1) [19].

**Table 1:** Impact of different factors on stability of “radiomics,” adapted from Timmeren et al. [19]

	Robustness		Reproducibility	Classification performance	
	Image acquisition	Reconstruction	Segmentation	Post-processing	Feature extraction
MRI	Field strength, sequence design, acquired matrix size, field of view, slice thickness, acceleration techniques, vendor, contrast timing, movement	Reconstructed matrix size, reconstruction technique	Manual/semi-automated/automated, 2D or 3D, ROI size	Image interpolation, intensity discretization, normalization	mathematical formula, post-processing platform
CT	Tube voltage, milliamperage, pitch, field of view, pixel spacing, slice thickness, acquisition mode, vendor, contrast timing, movement	Reconstruction matrix, slice thickness, reconstruction kernel, reconstruction technique			
PET	Field of view, pixel spacing, slice thickness, injected activity, acquisition time, scan timing, duty cycle, vendor, movement	Reconstruction matrix, slice thickness, reconstruction technique, attenuation correction			

## AI applications in spine imaging

AI enhancements are expected to have the potential to impact any industry, according to Gartner’s latest Hype Cycle for Emerging Technologies from 2020 [21]. AI techniques are already being used both in research settings and for commercial purposes for different tasks of routine workflows in spine imaging. Similar to approaches for categorizing AI-suitable tasks in spine surgery [9], the impact of AI may also be categorized into different spheres of action for (spine) radiology.

A recent literature review pictures a setting where ML and AI can improve overall diagnostic quality, e.g., by avoiding and quantifying reports [22]. However, potential efficiency opportunities go far beyond existing “imaging reads”–based workflows. The proposed current AI-driven transition from an “image reads” product to a “value chain” product providing high-quality information and holistic service will most likely be affected at every step of a “request to report” workflow. In addition, challenges of technical and legal nature, and challenges in adoption and uptake, necessitate the development of a clear strategy to move from a “reading images” mindset to a “complete value chain” mindset (i.e., a mindset of “creating and organizing information for greater accuracy, faster speed, and lower cost in medical decision-making”) [2].

**Fig. 1:**
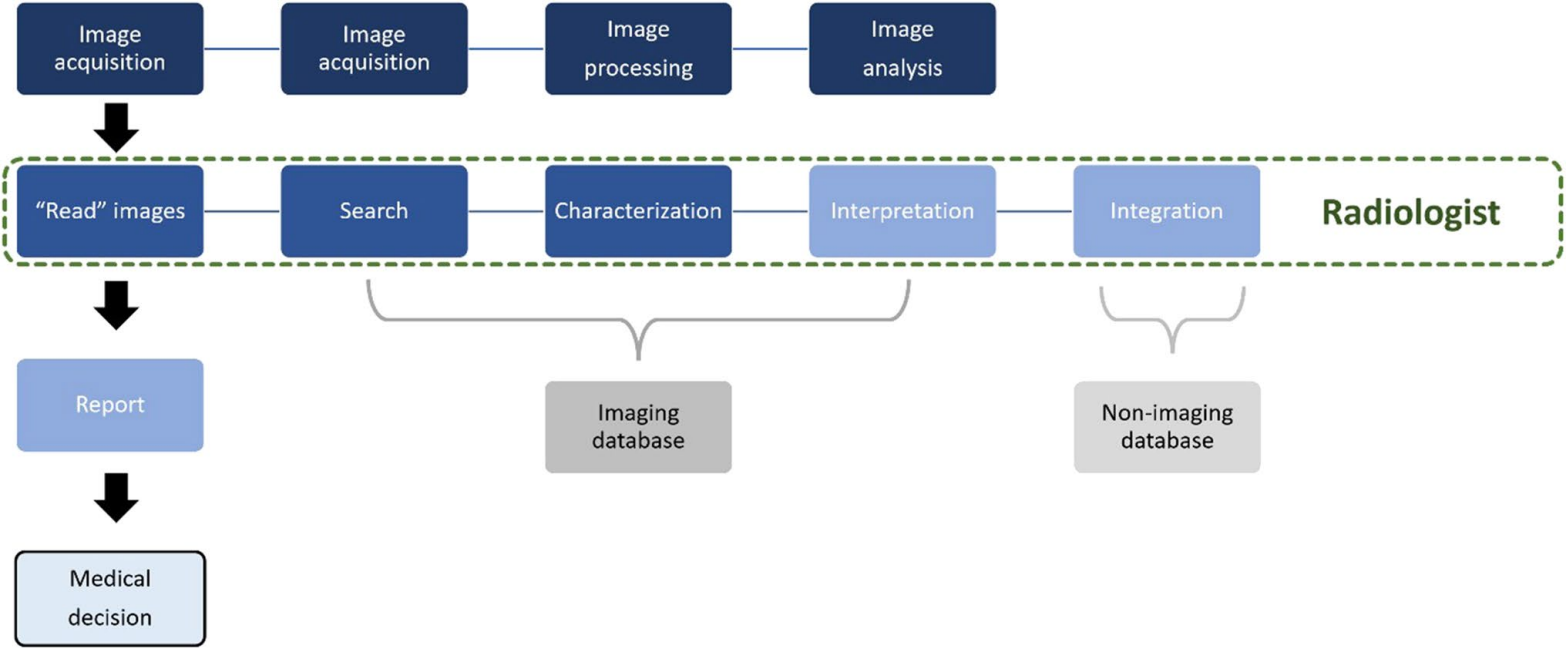
The radiology value chain, as described by Enzmann [2]. The main components of a classical complete radiology service model are image acquisition, “read” images, report, and medical decision

Hence, this section aims to describe the applications of AI for spine imaging–related tasks systematically, listing applications along a modified version of the value chain represented in Fig. 1.

### Patient selection and pre-examination workup

In addition to the four main stages of a value chain as described by Enzmann [2], the authors of this article propose an additional element of radiology workflows prior to imaging that is (and will further be) affected by AI technology on one hand, and which has major impact on overall productivity on the other. Therefore, this section aims to provide examples of AI approaches that have already been investigated for the improvement of pre-examination tasks, e.g., in patient selection and scheduling.

#### Patient selection

In general, radiology is facing an increasing number of requests specifically for cross-sectional imaging. Hence, identification of appropriateness criteria of when to perform which imaging procedure is highly warranted. For example, Manta et al. investigated that in MRI of the hip, 32% of the requests were deemed inappropriate. MRI for workup of uncomplicated low back pain is considered a substantial driver of MRI overuse and leads to unnecessary health care expenditures as well as potential risks to the patient such as radiation exposure (if a CT or X-ray scan is then requested based on MRI results), or allergic and non-allergic reactions to MRI contrast agents [23].

Efforts to stabilize or reduce numbers of unnecessary referrals have been implemented by initiatives such as Choosing Wisely Canada, which discourages spine imaging for uncomplicated low back pain [3]. As a response to similar campaigns, clinical decision support systems may help with detecting unnecessary referrals. So far, the first collaborations have shown positive results in implementing ML algorithms to enhance clinical decision support tools in this task [3]. For example, a retrospective study showed a significant decrease of imaging rates for lumbar MRI in low back pain by almost one-fourth, after staged implementation of a clinical decision support tool [24].

#### Optimized scheduling and examination protocoling

An important step in the value chain prior to protocol selection is the efficient scheduling of appointments. Recently, researchers applied ML techniques to predict failure of outpatients to attend scheduled hospital appointments. Predictions had considerable precision, and hence the ability to reduce costs in a generalized model [25].

Correct protocoling is a major discriminator in allowing for an efficient and professional workflow. The use of contrast media has limited value for several clinical questions, but it is necessary for a narrow spectrum of diseases, e.g., bone tumors. The protocolling task is time-consuming and often not trivial, and having it performed by radiology trainees might result in wrong protocol choices. A recent investigation used a deep learning–based natural language approach and aimed to determine the necessity of contrast media from free-text clinical indications of MRI referrals. For cases with perfect inter-reader agreement (100%) between two radiologists, the system showed an agreement of 90% [26]. On the other hand, AI may also help in automatically gathering and filtering patient risks like contrast media allergies or implants from the hospital record system.

### Image acquisition, processing, and analysis

#### Noise, motion, and other artifact reduction

Image noise reduces image quality, and noise correction is a routine step in image post-processing. However, different relations between noise and specific acquisition and reconstruction parameters are known and can therefore be anticipated. Hence, apart from traditional noise reduction strategies, e.g., scan time reduction in MRI, radiation dose reduction in CT, or more sophisticated statistical models, AI techniques have recently been investigated for noise reduction. To achieve this, mappings between noisy and noiseless images were trained, using natural images as well as images intentionally modified with added Gaussian noise of a defined range [27]. Subsequently, noise-free reconstructions from a noisy test image were successfully generated for MR, CT, and PET data [28–30].

MRI is prone to motion artifacts due to comparably long acquisition times. While several methods of motion correction exist, DL-based solutions are sometimes preferred, as they can correct for motion during post-processing without the need of input information regarding degree or type of motion-induced image distortion [27].

#### Scan time reduction

Generally, tradeoffs must always be made between spatial resolution, signal-to-noise-ratio, and scan duration [27, 31]. Reduced image quality is sometimes accepted in case of minor impact on diagnostic yield, since current routine post-processing includes different techniques for improving overall image quality, and therefore, the overall effect on image quality is minimal.

In terms of the reduction of image noise, AI (and specifically DL) algorithms are now able to perform mapping from lower-quality reconstructions to higher-quality reconstructions after an initial training by input data of full resolution. Thereafter, application to additional unrelated data is of comparable speed when compared to routine iterative reconstruction methods [32].

Recently, a technique of “virtual” or “synthetic” imaging has been investigated by different groups, where pre-trained CNNs later generate images that have not been actually acquired. For example, a generative adversarial network was able to create authentic synthetic MR images from CT data hard to differentiate from true acquired imaging data [3]. Moreover, Jans et al. proved that “synthetic CT” images of the sacroiliac joints, which were artificially created based on acquired MRI data, improved detection of structural bone lesions in patients with suspected sacroiliitis, compared with T1-weighted MRI [33].

A comparably advanced and very promising approach also for spine imaging has recently been published by Kleesiek et al., who demonstrated good diagnostic performance of an algorithm that generated virtual contrast-enhanced images from native brain MRI of tumor patients and healthy controls [34].

#### Spine labelling

As previously described, different ML methods are being used for labelling tasks throughout all medical specialties.

In imaging of the spine, a comparably redundant and trivial job is labelling of the respective vertebral level of a certain vertebral body or intervertebral disc from different images, such as radiographs, CT, or MRI data.

**Fig. 2:**
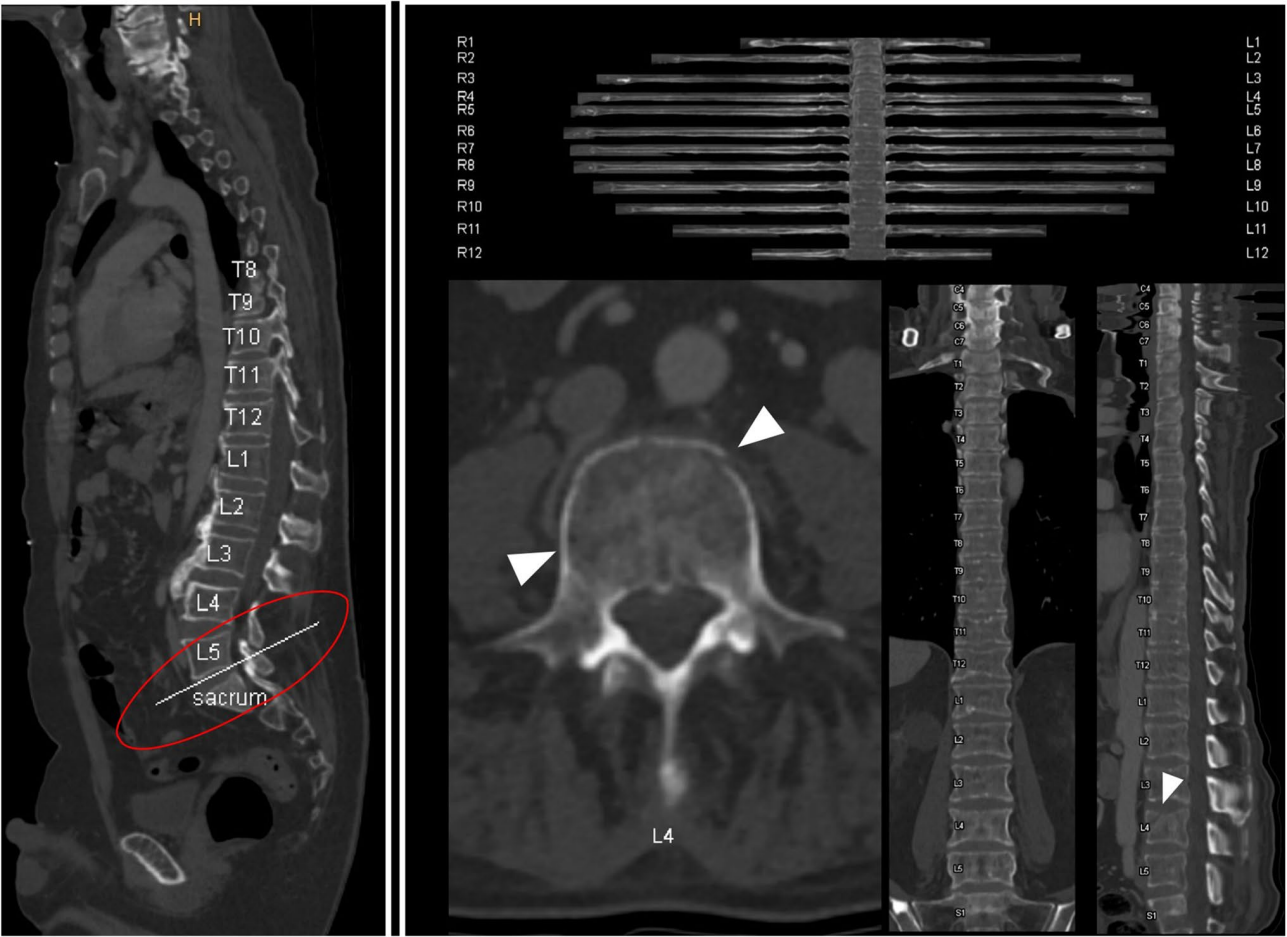
Routinely implemented automated image processing in spine imaging. Complex reconstructions of the whole spine are already implemented in clinical workflows for degenerative disease as well as in the trauma setting. The left part of the image shows automated output for spine labelling which allows for angle-corrected axial analysis of the vertebral structures (e.g., joint facets). Correct angulation for L5/S1 joint was detected (circled in red). The right part of the image summarizes standard workup of the spine and ribs with stretched multiplanar reconstructions as routinely done in trauma patients at the authors’ institute. Whereas ribs can be easily noted as normal, the compression fracture of the fourth lumbar vertebra (white arrowheads) is not only easy to detect, but can also be rapidly assessed with regard to complicating factors, e.g., spinal stenosis or instability. Images were acquired in Siemens syngo.via (syngo.via VB30A Bone reading, Siemens Healthineers, Erlangen, Germany)

Moreover, labelling and identification is usually the very first step in developing further automated algorithms for the purpose of disease detection or classification. Whereas labelling of spine level has already been implemented into several program suites of classical picture archive and communicating systems (Fig. 2), further research investigations have focused on determining the location of each intervertebral disc centroid in spinal MRIs. Schmidt et al., for example, achieved an error distance of 6.2 mm compared to a human reference standard [35].

Other groups even challenged algorithm performance in the aforementioned task by making it robust to datasets that included pathologies such as severe scoliosis or other deformities as well as presence of fixation devices, and received similar localization errors [36]. In another investigation, fully automatic labelling was also possible as a cross-modality tool [37].

#### Image segmentation

**Fig. 3:**
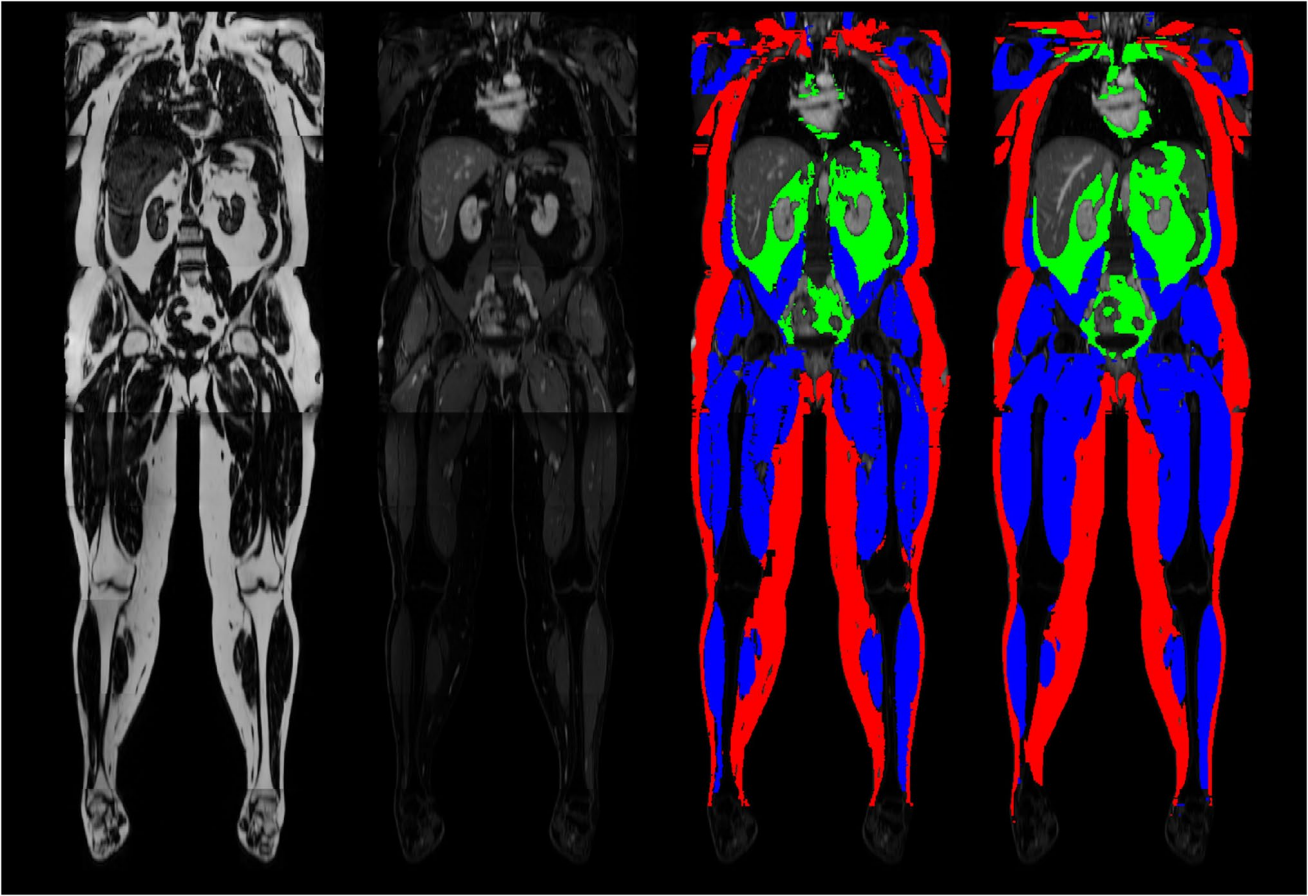
Example images from deep learning image segmentation in whole-body MRI. The images represent coronal multiplanar reconstructions of a T1-weighted Dixon-based dataset of a healthy individual. From left to right, fat and gadolinium-enhanced water sequences, as well as manually segmented “ground truth” segmentation mask and its automatic “pendant,” predicted by a deep learning–based MRI segmentation algorithm. Red, green, and blue areas represent the compartments subcutaneous adipose tissue, visceral adipose tissue, and muscle mass, respectively. All images unpublished own data, copyrighted by the authors

Image segmentation is defined as delineation of a prespecified region of interest (ROI) in image data. Usually, ROI borders represent specific anatomic or other semantic meanings. These ROIs are generally determined and differentiated by sharing similar or identical properties with other neighboring pixels, such as signal intensity in MRI or texture features in CT [38]. To date, manual segmentation is considered the most basic form, but also as the gold standard in segmenting images (compare Fig. 3). However, this task is tedious and trivial to the educated radiologist reader. In order to streamline segmentation processes and subsequent strategies of quantitative image analysis (such as TA), much effort has recently been invested in developing automatic segmentation algorithms, with variable success [39, 40].

With respect to MSK image data, implementation of fully automated segmentation algorithms is still tricky, primarily due to the complexity and possibility of variants in MSK cases.

Recent advances, however, were especially noted for spinal imaging in CT and MRI, where anatomy is comparably similar between different individuals. For example, Lessmann et al. presented a CNN with a memory function that is able to remember which vertebra was already classified. This method allowed for excellent segmentation accuracies with an average performance in terms of Sorensen-Dice coefficient of 0.94 (maximum 1) [41]. Furthermore, the performance of segmentation algorithms has also benefited from public challenges and open source databases that host annotated images [42].

Automated segmentation workflows also allow for novel opportunistic screenings. For example, different groups have recently proven that opportunistic screening for osteoporosis can be performed during CT imaging. Bone mineral density can be estimated both from standard 3D images of the trunk in unenhanced routinely CT [43] and experimentally from localizer data in photon-counting CT imaging [44].

#### Spinal and peripheral nerve segmentation

**Fig. 4:**
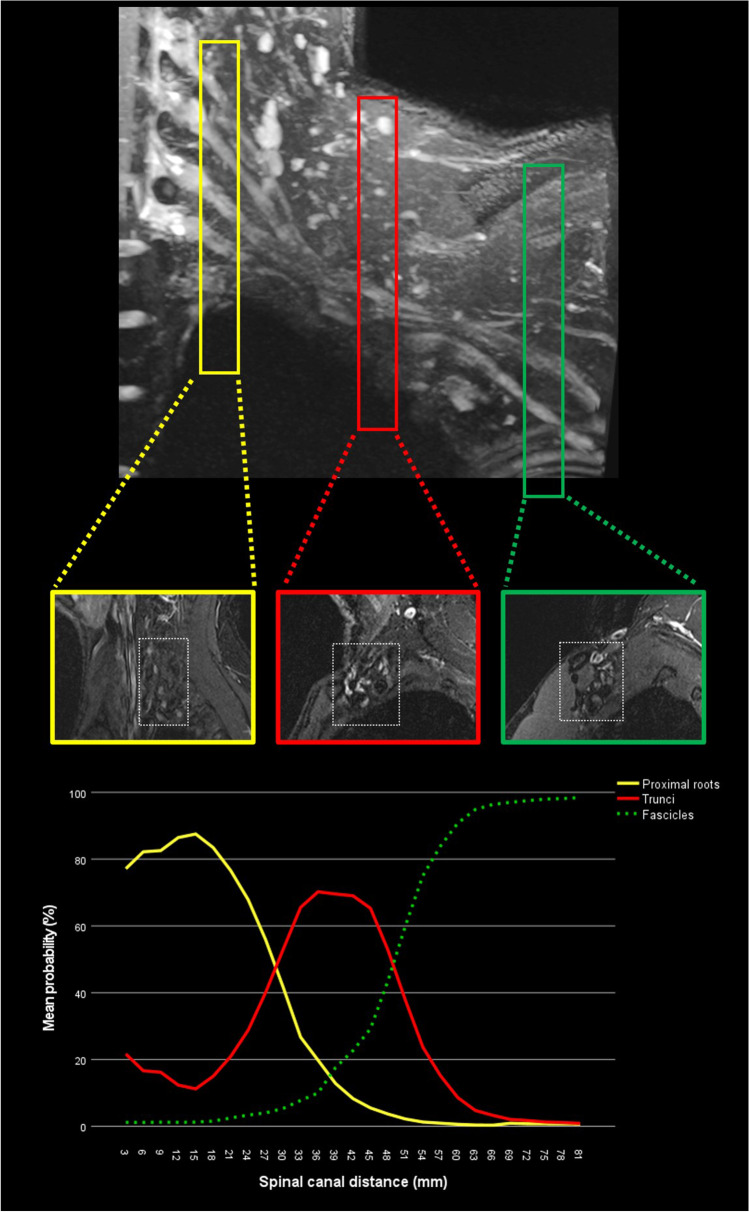
Algorithm performance expressed as probabilities of nerve position as either root, trunci, or fascicles in sagittal MR images of the brachial plexus (own unpublished data). Image numbers increase from medial to lateral, beginning at the cervical spine (3-mm slice thickness)

Recent publications have shown successful segmentation of peripheral nerves with similar accuracy and greater speed than manual segmentation [45]. This is especially relevant for any subsequent extraction of quantitative image data, e.g., diffusion tensor imaging that may help in diagnosing neuropathies. For example, in a research setting, the ischial nerve was fully automatically segmented in less than 1 s, compared to a manual segmentation time of 19 min. In a separate investigation by the current authors (unpublished data), it was furthermore possible to establish an AI-based workflow for automated detection of nerve position (root, trunci, fascicles) from paravertebral to a more peripheral sagittal image of cervical spine and brachial MR neurographies (refer to Fig. 4). Despite this being a work in progress, the authors of this review plan to implement a prediction function into a clinical workflow in a special hanging protocol for MR neurographies.

### “Read” images, image interpretation, and integration

#### Pathology detection and classification

In contrast to ever increasing imaging studies, the availability of on-site radiologists, especially in regional hospitals, is often limited. Therefore, automated detection of frequent pathologies, especially in common projection radiography, will help maintaining efficient patient management in hospitals without constant radiologic services. A series of investigations has been published with focus on diagnosis of spinal disorders in the various radiologic modalities [46, 47]. A recent systematic review by Azimi et al. has identified more than 40 studies that implemented NNs for diagnosis of spine disorders. For example, fracture detection by an algorithm is generally possible at comparable accuracy as orthopedic readers for most regions, including vertebral fractures [47]. Several other tools exist which focus on well-defined easy tasks, such as automatic measurement of spinal deformities by utilization of a neural network [48]. In addition, more sophisticated jobs have also been successfully accomplished by AI tools so far: Chmelik et al. trained a DNN for the detection of metastatic spinal lesions in CT regardless of acquisition parameters, by implementing patient- and protocol-dependent preprocessing. The first results indicate good accuracy, with sensitivity values of 0.8 and 0.92 for lytic and sclerotic lesions, respectively, provided the lesions have a minimum size of 1.4 mm^3^ [49]. Another group proved comparable accuracy between a DNN and three expert readers when differentiating between tuberculous and pyogenic spondylitis. The algorithm’s accuracy, expressed as area under the curve value, was 0.802 and was not significantly different to the pooled readers’ accuracy of 0.729 [50].

An even more advanced approach was recently published by Lewandrowski et al. [51]. The group developed a CNN-based algorithm, where class definitions for pathologies (e.g., canal stenosis) were extracted from the radiologist report after a 3D-model of the lumbar spine was fitted to each of the patient’s MRI prior to multisequence training. Preliminary results revealed comparable accuracy to a radiologist report.

In patients with multiple sclerosis, excellent performance is achieved for automatic lesion detection in the spinal cord. One example is Gros et al.’s investigation, where in addition to lesion detection, very accurate segmentations of the lesions were achieved (i.e., 95–98% similarity expressed by the Dice coefficient) when compared to that by a human reader [52].

#### Clinical translation and outcome prediction

**Fig. 5:**
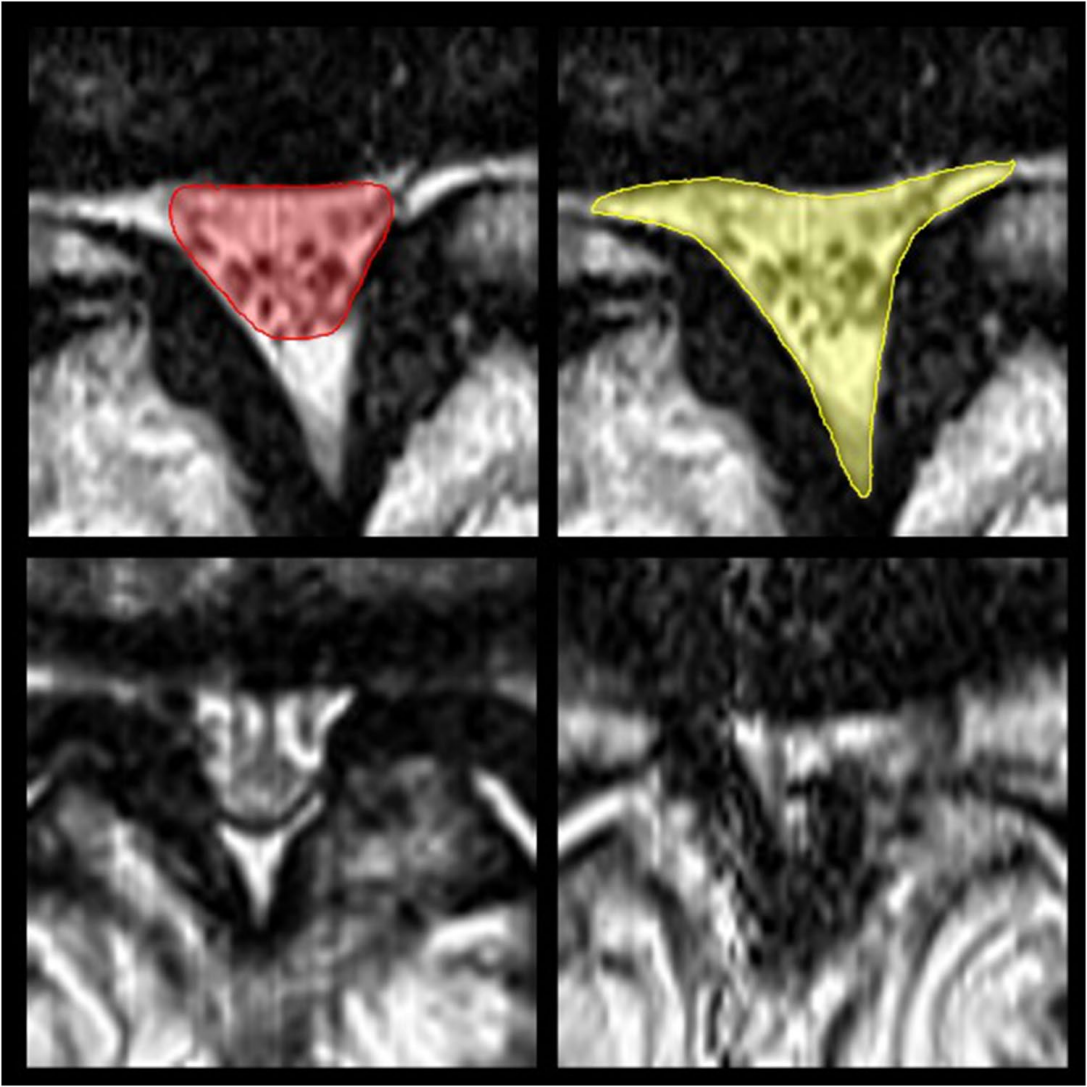
Quantitative (top row) vs. qualitative (bottom row) assessment of lumbar spinal stenosis severity. Texture analysis (TA) proved excellent reproducibility and objectivity regardless of whether only the central spinal canal was assessed (top left, red), or if instead the epidural sac and lateral recesses (top red, yellow) were included for measurements of the cross-sectional area. Moreover, TA outperforms qualitative approaches that differentiate between severe (bottom left) and extreme cases with epidural fat obliteration (bottom right)

Several studies have aimed to predict outcome parameters of spinal surgery by means of AI [53–55]. With respect to spine imaging, a series of studies has investigated the correlation between imaging and clinical findings as well as long-term outcome in a clinical cohort study of patients with lumbar spinal stenosis (LSS). For example, in a large clinical cohort of LSS patients, a series of studies revealed correlations between image and symptoms that were imperceptible to the reader’s eye with qualitative reading methods: Mannil et al. applied TA on the paraspinal muscles, where quantitative discriminators of muscle quality correlated with clinical disability questionnaires. A related study of this cohort similarly demonstrated increased accuracy and reproducibility of ML-supported TA for grading of LSS, compared to qualitative assessment (Fig. 5) [56–58]. Furthermore, TA was able to outperform advanced qualitative scores that take the compression of the epidural fat into consideration, as proposed by Schizas et al. [59]. In a related study, it was furthermore possible to predict fracture risk prospectively from post hoc TA of the vertebral bodies, derived from routinely acquired CT data of the trunk for other clinical reasons [60].

### Report

As speech-to-text recognition has revolutionized the workflow of radiologic reporting, AI has and will have the potential to enhance reports in numerous ways. Over the past few years, the use of structured reports has become increasingly popular in clinical routine [61]. AI has been proven to correctly generate structured reports from free text by using natural language processing (NLP). Additionally, investigations have shown use cases where a live algorithm could add fracture-related clinical knowledge in order to enhance a radiologist’s recommendation for further patient management. Other groups, as described, e.g., by Tan et al. demonstrated the ability of NLP for extraction of relevant text findings related to low back pain on reports of MRI and X-ray images [62]. This may also be helpful for future acceleration in retrospective research when looking for a specific cohort or data population.

### Medical decision

As a consequence of the aforementioned efforts of the past time, medical decision support tools are gaining increasing popularity in clinical routine, also in spine disease. One example is the recent “Nijmegen decision tool,” which recommends surgical treatment, conservative treatment, or no intervention at all in the setting of chronic back pain and is based on multivariate prediction of the patient’s health [63]. However, the majority of similar decision support tools are under development or in pre-clinical phases, as they require large clinical-radiological databases.

## Limitations and challenges of AI-based products

The tremendous recent achievements in medical imaging that were driven by AI have been answered by a very receptive radiology community with comparably few concerns regarding quality and validity of available AI tools in daily routine [64]. Nonetheless, the rapid progress in this research field has also fueled economic interest of private companies that have started offering AI-based clinical products for sale, while legal frameworks for medical product authorization in Europe and the US appear to have not adopted equally fast [65].

Therefore, recent guidelines have been proposed by Omoumi et al., aiming for a “voluntary” standardization in terms of technical, financial, quality, and safety aspects in the evaluation of AI medical products. The so-called ECLAIR guidelines are the first ones which aim to summarize all aspects affecting all relevant stakeholders, and which propose strategies for radiology practices regarding how to properly assess AI tools [66].

Apart from economic and quality considerations, AI tools have pushed into fields with only limited availability of legal boundaries. Sharing medical data for research purposes only is already a complex subject where individual privacy rights have to be weighed against potential benefits for society as a whole. However, AI data analysis of medical imaging data brings up new aspects of classical informed patient consents, as algorithms often require large amounts of sensitive image data for training and test purposes of an algorithm. Currently, we are experiencing a paradigm shift from traditional “informed consent” towards broader forms of consent (i.e. “broad consent,” “opt-out,” or even “presumed consent”) [64, 67]. This makes careful anonymization even more crucial a prerequisite for big data analyses. Recent investigations have demonstrated that traditional name anonymization in the DICOM header might not be sufficient in the future, as trained models that are publicly available can sometimes be misused to trace back to patient-related input data [68–70]. Safer and more up-to-date strategies include utilization of containerization and blockchain technology [64].

## Picture this…

Mr Johnny Spine wakes up on his waterbed in the late morning of the 1 of April in 2040 after an extensive dance performance on his 40th birthday party and experiences marked pain in his lower back radiating to his right greater toe. He consults his digital family physician on his mobile device, and based on his complaint-descriptions and personal data, the app comes up with a possible diagnosis of acute disc herniation including differentials. Mr Spine consents to do the suggested MRI examination of his lumbar spine, after the indication and referral has been digitally reviewed by his health insurance. The app suggests an appointment at the nearest radiology service, taking into account patient data, personal preferences, and local logistic conditions, including public transport availability, opening hours, and radiology hardware on site. After definite approval of the appointment by Mr Spine, respective imaging modality and scan protocol are suggested automatically based on a large-data DL algorithm, allowing to optimize patient throughput, scanner up-time, and costs of consumables on site. After being reviewed by the radiologist on call in the central reporting room of the group, this information is also communicated to Mr. Spine. When he arrives at the radiology facility, Mr Spine is asked by the technician to change into the functional clothes with integrated wearable-MR surface coils that have already been prepared for him based on physical phenotype data. The technician takes him into the open 3 T MR scanner at the exact accorded appointment time and positions him in a seated position. When pushing the start button after confirming correct patient and scan data, the MR scanner fully automatically acquires a set of imaging data of the lumbar spine. Also, the sacroiliac joints are included, as the AI on-site detects subtle subcortical abnormalities on the fly and triggers additional scan coverage. After about 5 min, Mr Spine is taken out of the scanner, asked to remove his MR clothes, and is free to leave the facility. In the meantime, images have been evaluated by a CNN algorithm assessing in detail every segment of the spine and producing standard reporting including specifications of the main findings in each segment and in relation to the actual problem. Virtual contrast-enhanced images were additionally post hoc–generated upon request of the supervising and signing radiologist, in order not to miss subtle inflammation as was suggested by the AI triage tool. All findings on the entire image stacks when reviewed by the radiologist are automatically and visually marked in a summary series that is also accessible for viewing via a password-restricted online access. As anticipated, a small subarticular disc extrusion at the level L5/S1 is diagnosed. SI joints are found normal. Mr Spine gets the report directly on his mobile phone in addition to — upon request — further recommendations for therapy. When looking up the images via online access at home, a fully digital radiologist explains the different anatomic landmarks and visual marks left by the AI and reviewed by the true radiologist. A patient cannot only ask questions but also request further information on specific findings and their clinical impact. When discussing the report with his treating physician, Mr Spine is wondering whether his overweight condition could have had an effect. The physician has full access to an online storage of all relevant images and patient history as priorly consented by Mr Spine. In addition, an orthopedic surgeon is consulted by the treating physician with full online access to all patient data. In order to comment on indication and suitability for surgery with respective prognosis, the orthopedic surgeon requires post hoc body composition and muscle quality analysis of the abdominal compartment from the same MR scan, using larger field-of-view reconstructions. Based on the analysis he puts, Mr. Spine on a strict low-carb diet as his phenotype seems to be prediabetic. Also, bone quality seems to be low for age, based on automatic radiomics analysis of the vertebral bones. Calculated probability scores for improvement based on imaging findings and conservative treatment are high, and the orthopedic surgeon therefore recommends conservative treatment. Mr Spine is convinced he can do it, strongly counting on his personal physiotherapist app that also came along with the image report from his radiologist and therapy recommendations from his treating physicians.
